# A research synthesis on successful educational practices and student outcomes for physical education in schools

**DOI:** 10.3389/fpsyg.2023.1280871

**Published:** 2023-10-02

**Authors:** Juan He, Hongli Yu, Man Jiang, Marcin Bialas

**Affiliations:** Department of Sport, Gdansk University of Physical Education and Sport, Gdansk, Poland

**Keywords:** school education, professional development, educational interventions, policy, student performance, physical activity

## Abstract

Although successful educational practices (SEPs) in higher education institutions have well-established student outcomes, the vast majority do not meet physical education standards in schools. Despite the promising nature of policy initiatives supporting schools, there is scant evidence of how these SEPs affect student outcomes. This review aimed to determine the status of the literature and the type of evidence regarding school SEPs. Several studies have demonstrated that these SEPs contribute directly or indirectly to improving student outcomes. Three objectives were examined and synthesized in our review of SEP research findings. The first goal is to identify different types of impacts on students in schools. The second goal is to provide educators, principals, and policymakers with a unified and comprehensive framework. Lastly, we provide suggestions for future SEP research. The review identified 45 studies that met our inclusion criteria. Our reviewed studies documented impacts on the individual level. It encompasses both students' instrumental abilities and their sense of self-esteem and motivation. Secondly, improving interpersonal relationships, reducing conflict, and increasing group cohesion are important components at the group level. Finally, there are factors at the community level, including absenteeism reduction, parental involvement, and changes in attitudes toward school. Current research supports the effectiveness of successful school practices. It stresses the importance of implementing policies to maximize student outcomes. Finally, the review concludes by discussing findings implications and future research directions.

## 1. Introduction

Physical inactivity is the 4th leading cause of premature death globally (Yu et al., 2022). The World Health Organization (WHO) recommends that children and adults participate in at least 60 min of physical activity (PA) per day and 150 min of PA per week, respectively (Woods et al., 2021). Promoting public health and preventing non-communicable diseases is recommended by health experts. Despite the numerous studies that cite the positive effects of PA (Yu et al., 2023), epidemiological research has concluded that the benefits are not widespread. It is estimated that only 28% of adults and 81% of children and adolescents meet the eligibility criteria for this initiative (Hardy et al., 2017; Guthold et al., 2018). Adolescence is consistently characterized by declining PA levels (Dumith et al., 2011; Hallal et al., 2012; Kim et al., 2012; Cooper et al., 2015). Their PA behaviors are influenced by their childhood experiences (Telama et al., 2005; Telama, 2009; Murphy et al., 2016; Hardie Murphy et al., 2017). It has also been demonstrated that boys are more physically active than girls (Murphy et al., 2016; Hardie Murphy et al., 2017). This high level of inactivity among children requires effective measures to reduce it.

The inactivity challenge is well documented in the literature in terms of solutions (Yu et al., 2022). The literature also emphasizes a multi-level response based on an ecological perspective (Liu et al., 2022; Arif et al., 2023; Hira et al., 2023) that considers factors related to the individual, environment, and policy. Tobacco products have been successfully eliminated by addressing all these levels (Woods et al., 2021). Policy interventions should focus on reducing lifestyle-related diseases associated with physical inactivity by targeting upstream determinants of health behavior (Lakerveld et al., 2020). One such initiative is the WHO global action plan for preventing and controlling non-communicable diseases (Hardy et al., 2017). The initiative aims to reduce inactivity prevalence in the population by 10% in the future (Woods et al., 2021). An action plan developed by WHO recently outlines several policy initiatives related to PA. An integrated pedagogical approach and a systems-based approach are included in this program. This makes it imperative to understand the underlying causes of inactivity. We can only hope this will improve health and promote PA. Furthermore, it is imperative to combat the global epidemic of obesity, climate change, and undernutrition (Swinburn et al., 2019).

Most kids and teens spend so much time in school, making it an important environment for their development. While there are recommendations for whole-school approaches to PA promotion (Milton et al., 2021), very little research has been conducted on the effectiveness of PA policies in schools. Whole-school methods rely heavily on policy decisions. Several factors influenced children's PA in one study (Lounsbery, 2017). It discusses the existence or absence of laws and the nature of those laws (required or encouraged). Children's participation in PA can be significantly influenced by the extent to which it is carried out. Some reports examine the various levels of education and the key decision-makers at each level. It was comprised of representatives from the national, regional, district, and school principals and teachers from the classroom. It also provided an overview of the different levels and key policymakers at each level. Though PA policies are generally well-intended, implementing them is not always straightforward. Policies related to PA differ greatly in terms of specificity, funding, implementation, and accountability (Lounsbery et al., 2013). In order to improve PA within the school setting, it is necessary to investigate the current evidence status.

The paper defines successful educational practices (SEPs) as “decisions, plans, and actions of government agencies and organizations aimed at achieving specific educational health goals at the national, regional, or local levels, regardless of whether they are directly or indirectly applied” (Lakerveld et al., 2020). SEPs aim to modify systems rather than individuals to create conditions that are conducive to the reduction of non-communicable diseases such as obesity by promoting conditions and interventions that can be implemented together. The main distinction between policy and other types of programs or environmental interventions is that they lay the groundwork for generating solicitations, developing, financing, and implementing other interventions (Gelius et al., 2020).

In educational settings, SEPs have been advocated in several frameworks as a tool for promoting health. Although these frameworks are primarily concerned with a broad definition of health, they offer a valuable conceptual starting point for investigating policy's potential direct and indirect effects on PA. To help policymakers at national and subnational levels in establishing and developing school policies, the WHO published a school policy framework based on the food and PA strategy (Woods et al., 2021). As a result of this approach, it was emphasized that children are not immune from inactivity. It was urged to implement changes immediately to improve PA levels among children. This activity aimed to develop and implement policies promoting PA among children. A recent shift has occurred toward identifying all the components of a whole school PA strategy through the development of the Creating Active Schools Framework (Daly-Smith et al., 2020). In this theoretical stance, schools are viewed as complex adaptive systems, with the concept of “active school” at the center of the institution's guiding principles. It is commonly understood that an “active school” is one that actively promotes PA for its students by bringing together its numerous constituents (students, parents, administration, faculty, and the surrounding community).

Historically, SEP have been pursued with the ultimate goal of improving student outcomes (Woods et al., 2021). Similarly, physical education (PE) emphasizes the holistic development of students as well as traditional academic disciplines (Dumith et al., 2011). In the realm of PE, it is pertinent to examine how SEP can have a profound impact on student outcomes, as it examines how strategies, methodologies, and approaches used in PE instruction impact students' overall development and achievement. In addition to promoting health and fitness, PE is often considered an integral part of a well-rounded education. Besides fostering physical health, it also fosters mental, social, and emotional well-being through a variety of activities and learning experiences (Cooper et al., 2015; Hardie Murphy et al., 2017; Lakerveld et al., 2020). It has been shown that these pedagogical practices have a profound effect on students' ability to perform physically (Lounsbery et al., 2013; Murphy et al., 2016). They also affect their sense of self-esteem, their abilities to work as a team, and their attitude toward PA and wellness throughout their lives (Hardie Murphy et al., 2017; Gelius et al., 2020). Our study will examine the impact of different approaches on student engagement, skill development, and overall well-being. We will examine empirical evidence, case studies, and scholarly research. Education stakeholders, policymakers, and educators must understand this relationship to plan for the future of education. The importance of lifelong PA and its positive impacts on both physical and mental health are becoming increasingly apparent around the world. SEP in PE has an even greater role to play. Educators can promote learning, growth, and achievement by identifying effective strategies and best practices that are tailored to meet the needs of diverse students.

It has been more than a decade since researchers began researching the SEP method and its use in the classroom (Dumith et al., 2011). Studies at the study level support the hypothesis that students participate in these school-based initiatives. These results have already been published in scientific journals (Morla-Folch et al., 2022). It is possible to gain valuable information about how SEPs work and what effects they produce from the specialized study literature on SEP. Previous studies have shown that students' schooling, well-being, and social relations improve when exposed to positive influences (Woods et al., 2021). The varying outcomes for student participants in various programs have not been examined within a cohesive framework based on previous knowledge. An overview of the literature is presented here, synthesizing studies examining how SEPs impact students' academic and social performance. Our synthesis seeks to 1) demonstrate the various effects of SEP participation on students, 2) provide a cohesive and comprehensive description of SEP's impact on students, and 3) provide recommendations for future research.

## 2. Methods

We performed a research synthesis in response to our research questions (student outcome and psychological wellbeing), as explained by other researchers such as Cooper (2015). As a research synthesis, we used an integrated approach that focused on the impacts of SEPs on preschool and primary school students by examining the findings of a body of research through a specific question as reported by Compton-Lilly et al. (2021). Throughout the research process, we took a logical approach to research synthesis in different phases. In the first step, we searched pertinent scientific databases, as followed by Morla-Folch et al. (2022). Secondly, the retrieved literature was screened, and irrelevant literature was eliminated according to a set of inclusion/exclusion criteria as described in detail by Woods et al. (2021). Finally, we conducted a mixed-methods synthesis of the selected case studies, as followed by Heyvaert et al. (2013).

### 2.1. Search and selection of literature

**Figure 1:**
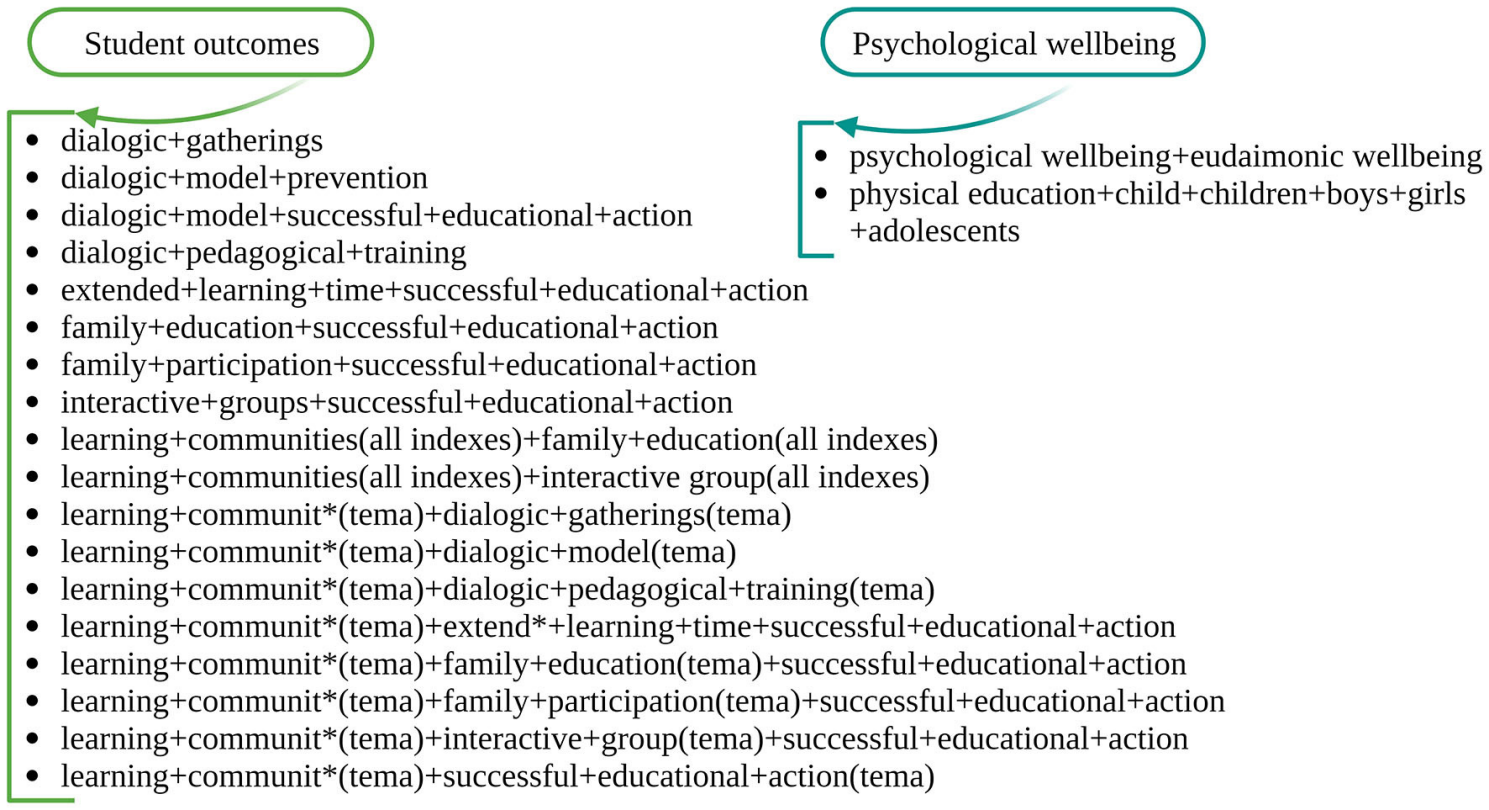
Two examples of search syntax used.

**Figure 2:**
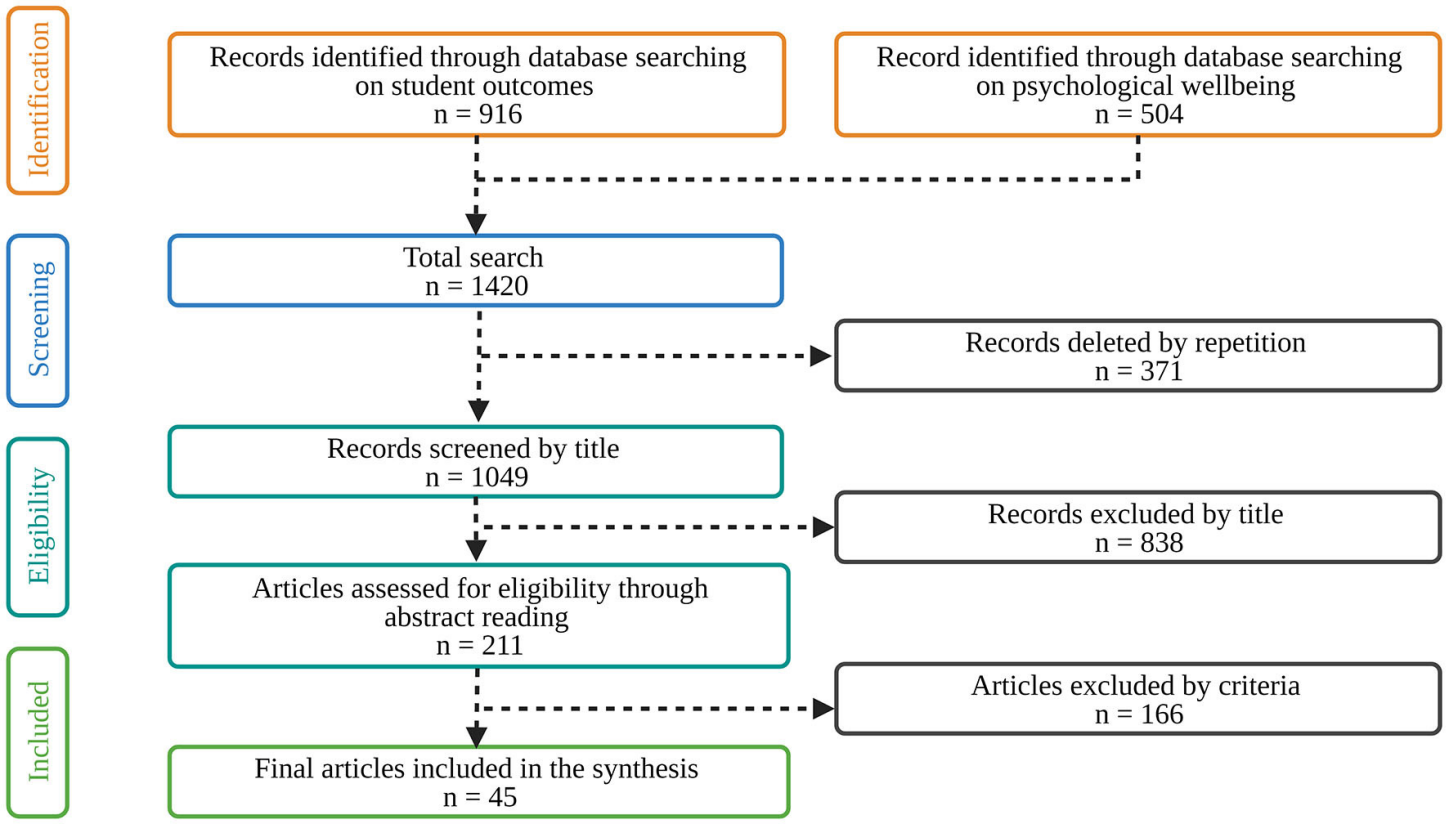
Flowchart of the search and selection process.

The literature was searched using a list of primary search terms (individually) compiled from our previous knowledge of SEPs (see Figure 1). As we expected these databases to provide comprehensive coverage, we systematically consulted three databases from 2000 onward: Web of Science, Scopus, and Scielo. All three databases were accessed using English terms. The search resulted in 916 records on student outcomes (Figure 2). Also, we searched these databases for psychological wellbeing and obtained 504 records. Our first sample of 1049 publications was obtained after removing duplicates. Based on the title and abstract read within each publication, we excluded irrelevant publications based on the researchers' structural review approach, as followed by Moher et al. (2015). A full-text review of the remaining 211 records was performed before the final selection was made. The following criteria were established for inclusion/exclusion based on our literature review objectives: Empirical studies focused on studying the impact of effective practices on preschool and primary school children's educational outcomes. As a result of careful reading of the full articles by the first three authors, a few more articles were excluded, resulting in a final sample of 45 articles (see Table 1).

**Table 1:** General overview of the articles included.

**No**.	**Authors and year of publication**	**Publication type**	**Institute location**	**Sample size**	**Categories**
1	Aubert, 2015	JA	Spain	3	IL; SSM; CCR; IR; SFC
2	Aubert et al., 2017	JA	Spain	6	IL; SSM; CCR; IR; SFC; AR
3	Bakir et al., 2017	JA	Turkey	60	IL; SSM; CCR; IR
4	Beaulac et al., 2011	JA	Canada	63	IL; SSM; CCR; IR; SFC; AR
5	Capllonch Bujosa et al., 2018	JA	Spain	300	CCR; IR
6	Connolly et al., 2011	JA	United Kingdom	55	IL; SSM
7	Costigan et al., 2016	JA	Australia	65	IL; CCR; IR; AR
8	Diez et al., 2011	JA	Finland, Lithuania, Malta, Spain, UK.	32	SFC
9	Diez-Palomar et al., 2020	JA	Cyprus, Italy, Spain, United Kingdom	418	IL
10	Flecha and Soler, 2014	JA	Spain	13	IL; SSM; SFC; AR
11	Garcia-Carrion, 2015	JA	Spain	7	IL; SSM; CCR; IR; SFC
12	Garcia-Carrion et al., 2018	JA	Spain	1	IL; SSM; IR
13	Garcia-Carrion et al., 2020a	JA	Spain	9	SFC
14	Garcia-Carrion et al., 2020b	JA	Spain	24	IL; SSM; CCR; IR
15	Gomez et al., 2014	JA	Spain	13	CCR; IR; SFC; AR
16	Grace, 2015	JA	South Africa	31	SSM
17	Gül et al., 2017	JA	Turkey	187	SSM; CCR; AR
18	Ha et al., 2017	JA	China	773	SSM; CCR; IR; SFC
19	Hankonen et al., 2016	JA	Finland	1,123	IR; AR
20	Hignett et al., 2018	JA	United Kingdom	58	CCR; SFC; AR
21	Ho et al., 2017	JA	China	664	IL; SSM; CCR; IR; SFC; AR
22	Huescar Hernandez et al., 2020	JA	Spain	102	IL; SSM
23	Kall et al., 2015	JA	Sweden	428	SSM; CCR; IR; SFC; AR
24	Karasimopoulou et al., 2012	JA	Greece	286	IL; SSM; CCR; IR; SFC; AR
25	Khalfaoui et al., 2020	JA	Spain	10	IL
26	Lopez de Aguileta, 2019	JA	Spain	2	IL
27	Lopez de Aguileta et al., 2020	JA	Spain	28	CCR; IR
28	Lubans et al., 2016	JA	Australia	361	SSM; CCR; IR; SFC; AR
29	Luna et al., 2019	JA	Spain	113	SSM; CCR; IR; SFC
30	McNamee et al., 2017	JA	USA	1,970	IL; SSM; CCR; IR; SFC; AR
31	Molina Roldan, 2015	JA	Spain	2	IL; SSM; IR
32	Noggle et al., 2012	JA	USA	51	IL; SSM; CCR; IR; SFC; AR
33	Padros, 2014	JA	Spain	15	CCR; SFC
34	Puigdellivol et al., 2017	JA	Spain	10	IL; CCR; IR
35	Roca et al., 2020	JA	Spain	5	SSM; CCR
36	Sifers and Shea, 2013	JA	USA	111	IL; SSM; CCR; IR; AR
37	Slee and Allan, 2019	JA	United Kingdom	100	SSM; CCR; IR; SFC; AR
38	Smedegaard et al., 2016	JA	Denmark	3,124	SSM; CCR; IR; AR
39	Standage et al., 2013	JA	United Kingdom	711	IL; SSM; CCR; IR; SFC; AR
40	Valero et al., 2018	JA	Spain	55	IL; SSM; CCR; IR
41	Valls and Kyriakides, 2013	JA	Spain	16	IL; SSM; IR; SFC
42	Villardon-Gallego et al., 2018	JA	Spain	442	IR
43	Villarejo-Carballido et al., 2019	JA	Spain	11	CCR; SFC
44	Wright and Burton, 2008	JA	USA	23	IL; SSM; CCR; IR; SFC; AR
45	Zubiri-Esnaola et al., 2020	JA	Spain	25	IL; SSM; IR

AR, Absenteeism Reduction; CCR, Cohesion and Conflict Reduction; IL, Instrumental Learning; IR, Interpersonal Relationships; JA, Journal Article; SFC, School, Family, Community; SSM, Self-esteem and Motivation.

### 2.2. Literature analysis

Several analysis rounds were performed on the literature. It was first necessary to conduct a thematic analysis to structure all the themes related to SEPs and their impact on preschool and primary school children. Our approach enabled us to summarize the key characteristics of a complex data set while maintaining a detailed description, as followed by Heyvaert et al. (2013). In order to identify salient themes related to our research questions, the first three authors (authors of the synthesis) read each article thoroughly independently, considering the risk of bias. To conceptualize and categorize the findings, we first immersed ourselves in an open coding first reading. Particularly, we sought to identify studies that focused on SEP's impact on students' performance. Our next step was to compile the results of our development round. We also formulated a common coding scheme and discussed and refined it.

**Figure 3:**
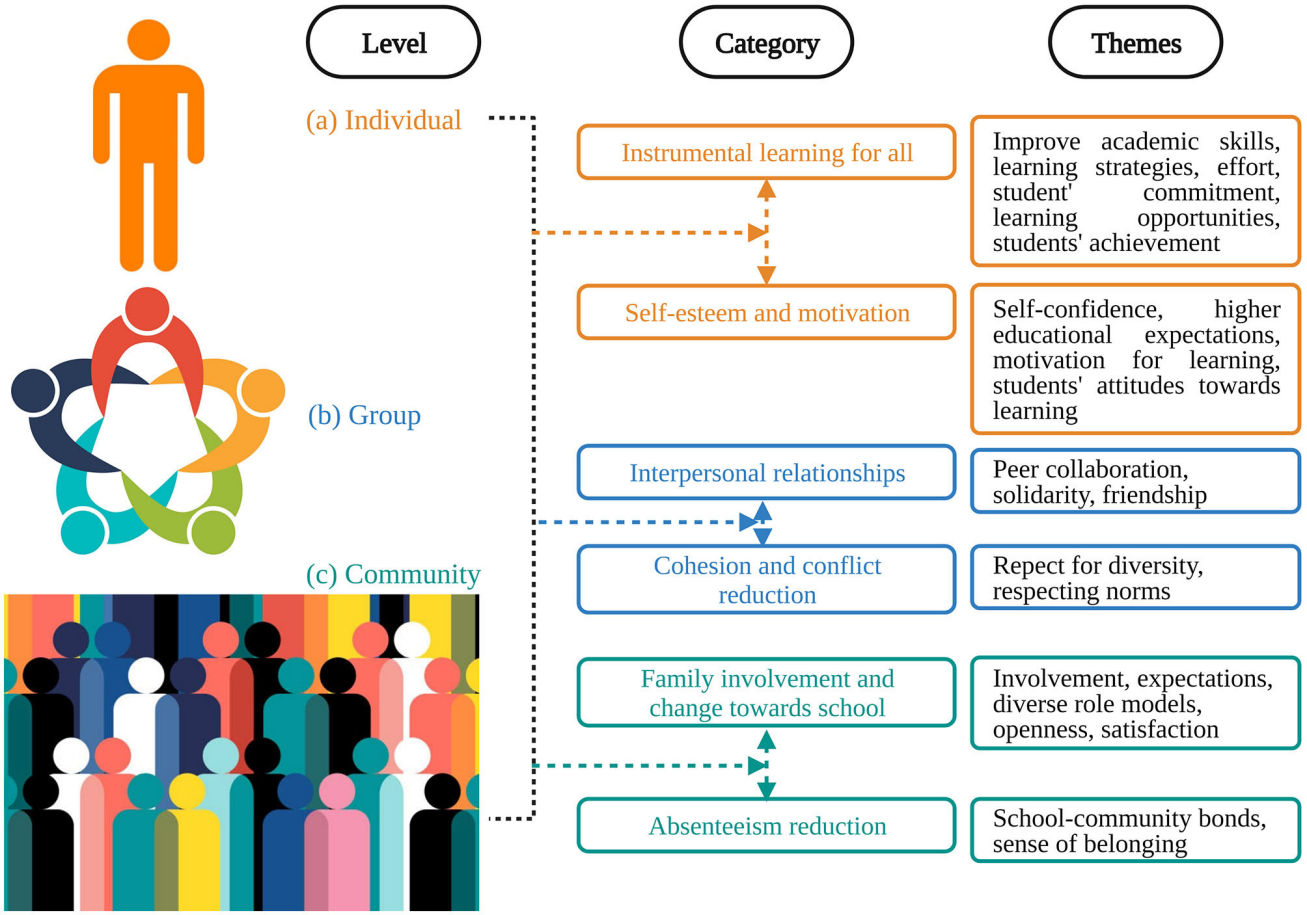
Themes and categories used in this study.

The second step involved the development of a codebook that identifies and describes the major coding categories (see Figure 3). At this point, we attempted to maintain a broad description of the context, as much as possible, to comprehend the full scope of each study, as mentioned by Doyle (2003). We have included additional descriptive information in the codebook to facilitate additional information extraction from each article. Reference numbers were assigned to each study publication year, publication type, institute location, sample size, and categories (Table 1). The information to be extracted from the primary studies was compiled in a spreadsheet in Excel using all the categories mentioned above.

Thirdly, we reread each study carefully a second time. The process of reading as a reviewer was initiated in this round using a defined coding scheme (Compton-Lilly et al., 2021). The data were extracted from the spreadsheet, and the descriptive information was coded according to analytical themes. Analytically coded themes were derived by extracting the exact words from the reviewed articles and coding relevant paragraphs of the primary studies into the agreed-upon category codes. Often, it was challenging to determine the selection and length of the selected text and quotations (Soilemezi and Linceviciute, 2018). Therefore, we agreed to select complete, meaningful paragraphs. We reached a consensus after discussing the complete classification of the coded paragraphs and the full coding by category (Table 1).

The findings were discussed, and individual, group, and community stories were created for each category (Doyle, 2003), highlighting the nuances and contrasts between successful practices at three levels: individual, group, and community. A full interpretation of our categorical scheme was created by combining all the pieces. It was our goal to distill the data into a coherent interpretation that encompassed more than just the parts that we had examined (Flemming and Noyes, 2021). The results were close to being written at this point.

## 3. Results

A dynamic and multifaceted relationship exists between SEP and students' outcomes in PE. This relationship is driven by the interactions between various factors contributing to students' holistic development. Several empirical studies and extensive research (Wright and Burton, 2008; Beaulac et al., 2011; Connolly et al., 2011; Diez et al., 2011; Karasimopoulou et al., 2012; Noggle et al., 2012; Sifers and Shea, 2013; Standage et al., 2013; Valls and Kyriakides, 2013; Flecha and Soler, 2014; Gomez et al., 2014; Padros, 2014; Aubert, 2015; Garcia-Carrion, 2015; Grace, 2015; Kall et al., 2015; Molina Roldan, 2015; Costigan et al., 2016; Hankonen et al., 2016; Lubans et al., 2016; Smedegaard et al., 2016; Aubert et al., 2017; Bakir et al., 2017; Gül et al., 2017; Ha et al., 2017; Ho et al., 2017; McNamee et al., 2017; Puigdellivol et al., 2017; Capllonch Bujosa et al., 2018; Garcia-Carrion et al., 2018, 2020a,b; Hignett et al., 2018; Valero et al., 2018; Villardon-Gallego et al., 2018; Lopez de Aguileta, 2019; Luna et al., 2019; Slee and Allan, 2019; Villarejo-Carballido et al., 2019; Diez-Palomar et al., 2020; Huescar Hernandez et al., 2020; Khalfaoui et al., 2020; Lopez de Aguileta et al., 2020; Roca et al., 2020; Zubiri-Esnaola et al., 2020) have examined various SEPs in PE and their impact on student outcomes. The search resulted in 45 articles meeting our inclusion criteria (Woods et al., 2021). Studies reported in the reviewed articles focused on developing interactive groups and dialogue gatherings involving literature. Geographically, most articles presented Spanish studies. Table 1 summarizes the descriptive characteristics of the articles reviewed. Most articles examined reported on a single case study and used qualitative research methodologies. Qualitative studies are based on a communicative methodology. This is where participants and researchers jointly engage in a dialogue to understand social reality. This is done by interacting with multiple and diverse voices. Data collection for qualitative studies uses a variety of methods. It includes classroom observations, in-depth interviews, semi-structured interviews, and focus groups. Students, teachers, parents, community agents, educational administrators, and school administration teams are included in their studies. The researcher and participant engage in a dialogue aimed at recognizing, reflecting on, and interpreting their daily lives in four qualitative studies (Molina Roldan, 2015). Six articles in the review use document analysis to examine test results, student outcomes, and school reports (Valls and Kyriakides, 2013; Flecha and Soler, 2014). Several studies reviewed incorporated classroom observations, communicative daily life stories, semi-structured interviews, and questionnaires into their mixed methods designs. There was an increased emphasis placed on qualitative data in these studies, while quantitative data supported the investigation or provided context for the event studies (Capllonch Bujosa et al., 2018). Researchers used cross-sectional data, self-reported questionnaires, and quasi-experimental de-signs in only three of the reviewed studies (Garcia-Carrion et al., 2018; Gutierrez-Fresneda, 2019; Huescar Hernandez et al., 2020). Our next step is to summarize our findings, based on three levels of analysis: individual, group, and community. We aim to provide information concerning successful practices' influence on students.

### 3.1. Individual level

Twenty-six articles discussed the effects of instrumental learning on specific learning outcomes related to specific subject matter (Table 1). The articles studied described improved learning outcomes for students. This phenomenon has been observed most frequently in the areas of mathematics and language (Flecha and Soler, 2014) and, to a lesser extent, in the field of language (Aubert et al., 2017; Lopez de Aguileta, 2019) or in the field of mathematics (Garcia-Carrion, 2015). Nevertheless, successful practices have been implemented in many other disciplines, including sports, English instruction (Zubiri-Esnaola et al., 2020), and religion (Garcia-Carrion et al., 2020a). According to the reviewed articles, national standardized tests or school test results were used to assess students' learning outcomes. Even though most of the articles studied focused on primary school children, one article (Aubert et al., 2017) revealed that preschool students have improved learning outcomes. A study found that after 3 months of PA, the reading and writing skills of 5-year-old children have improved (Aubert et al., 2017). Furthermore, the reviewed articles indicated that special education students benefit from attending school. It has been reported that SEPs impact these students. This paper contends that students can continue participating in the typical classroom environment, doing the same activities as their classmates (Valls and Kyriakides, 2013; Molina Roldan, 2015).

Results of school tests or national standardized tests were used to base studies findings in the examined articles. One article (Aubert et al., 2017) showed improved learning outcomes among preschoolers. Despite this, many of the reviewed articles were aimed at primary school students. PA improved the reading and writing abilities of 5-year-old children over 3 months (Aubert et al., 2017). Additionally, the articles reviewed indicate that students with special needs gain benefits from attending school. According to researchers (Valls and Kyriakides, 2013; Molina Roldan, 2015), students can successfully participate in the ordinary classroom by participating in the same activities as their peers.

The results of thirty studies indicate that SEPs encourage students to have high expectations, be self-confident and be confident in their learning abilities. Furthermore, studies have found that students participating in successful practices have a higher expectation of their performance in school (Garcia-Carrion, 2015) and of continuing their studies in the future (Aubert, 2015). This finding was supported by the reviewed articles, which accounted for increased self-esteem among the students. Multiple articles have reported that students from underprivileged backgrounds feel more confident because they have demonstrated their capacity to succeed. Students reported that they had gained more self-confidence from dialog gatherings (Aubert, 2015) and those immigrants had improved their self-esteem (Valero et al., 2018). Additionally, the reviewed articles demonstrate that SEPs increased students' self-confidence, enabled them to accomplish things they were unable to before, and in turn improved their academic performance. Researchers presented an analysis of two case studies based on biographical narratives (Aubert, 2015; Elboj, 2015). It shows that two minority students exhibited significant improvements in self-esteem and self-confidence.

### 3.2. Group level

Thirty-one articles were included that addressed various aspects of interpersonal relationships between students (Table 1). It has been observed in the reviewed articles that interpersonal relationships can be measured by the level of students' help (Garcia-Carrion et al., 2018), supportive relationships (Aubert, 2015; Aubert et al., 2017), cooperative attitudes (Diez et al., 2011), and solidarity (Valero et al., 2018; Villardon-Gallego et al., 2018). It has been proposed that foreign and local students together enhance interpersonal relationships by creating an environment that is equitable, positive, and non-stereotypical (Valero et al., 2018). It has been shown in various studies that students learn how to collaborate effectively, get to know one another and form better relationships as a result. Meanwhile, academics have provided evidence to support the view that gatherings provide a conducive environment for friendship by displaying the testimony of a student who explains that he was able to make friends through the meetings since they enabled him to exchange ideas outside of the classroom (Grace, 2015).

It has been shown that SEPs enable students to express their own opinions and emotions in a manner that enhances cohesion among students, fosters friendship among students (Aubert et al., 2017; Duque et al., 2020) and creates bonds of solidarity and mutual help (Villardon-Gallego et al., 2018). A study found that gatherings were associated with prosocial behavior among students (Khalfaoui et al., 2020). According to the authors, preschoolers can engage in flexible interactions by arranging the classroom dialogically, which promotes dialogic learning. Additionally, several articles indicated that students assist one another in solving learning problems. Scholars have found that students are willing to assist their classmates in learning their class content (Aubert et al., 2017). The study aimed to determine how students interact with their peers collaboratively. Study participants observed students instructing their peers on how to complete learning tasks, thus facilitating the consolidation of learning and creating a common understanding between both groups despite their cultural differences (Garcia-Carrion, 2015). There is a premise inherent in the gatherings that the “whole group completes the task” (Valls and Kyriakides, 2013), which motivates students to work together to resolve the problem.

There are 33 articles in the cohesion and conflict reduction category. These articles deal with issues such as improving the classroom climate, promoting cohesion through diversity, fostering respect, and preventing violence. According to the reviewed article authors, SEPs promote inclusion for all students, regardless of age, gender, cultural background, or physical background (Lopez de Aguileta et al., 2020). A scholar describes how a girl whose class-mates bullied her underwent a transformation using the biographical method (Aubert, 2015). An additional study focused on implementing a dialogic conflict resolution and prevention model. It was found that school coexistence improved because of the implementation of that model (Villarejo-Carballido et al., 2019). It was revealed through participants' quotations that the school promoted the concept of standing up for victims subjected to violence, which has led to students intervening when they witness violent situations and denying them (Villarejo-Carballido et al., 2019).

One article addressed conflict prevention in physical education classes by implementing conflict reduction strategies (Capllonch Bujosa et al., 2018). The authors implemented these SEPs and found that they significantly reduced the number of conflicts between students during the class. It has been demonstrated that cooperative games facilitate the development of teamwork abilities, conflict resolution, and the achievement of common goals (Capllonch Bujosa et al., 2018). According to the authors, these actions resulted in higher student engagement, greater compliance with norms, and fewer conflict situations. There are also some limitations to the studies reviewed. SEPs establish horizontal dialogue among all community members because of their implementation. Assembly organization and discussion of coexistence norms take up much time, but conflicts persist. A key aspect of the initiative is the involvement of the whole community. Investigators concluded that a lack of commitment by any school member hinders the achievement of objectives (Capllonch Bujosa et al., 2018).

### 3.3. Community level

The impact of family involvement initiatives on students was reported in 23 articles (Table 1). It has been reported in several articles that family involvement in education improves learning outcomes (Molina Roldan, 2015; Puigdellivol et al., 2017), learning motivation, and conflict resolution (Padros, 2014). Traditionally, underprivileged communities have been excluded from educational opportunities, making this issue even more acute. Research has indicated that school initiatives involving family members in the context of social exclusion positively impact students' perceptions of their school (Garcia-Carrion et al., 2018). Consequently, their academic performance is affected by perception change. Research indicates that students are encouraged to engage in school activities by their parents (Villardon-Gallego et al., 2018), and that when their parents value school, students are more likely to be enthusiastic about participating in school activities. Moreover, the papers reviewed indicate that parents are more likely to assist their children with their homework and school activities if they are allowed to participate actively in successful practices (Aubert et al., 2017; Garcia-Carrion et al., 2020b). Several researchers have raised the expectations of family members regarding their children's education (Aubert et al., 2017). This has resulted in more enthusiastic and motivated children participating in the educational process.

Family education has been found to improve the basic skills of family members in the selected articles. Among its components are reading and writing (Flecha and Soler, 2014) and digital literacy (Garcia Yeste et al., 2018). This resulted in better learning outcomes for students with better family skills. Several studies have found that family learning enhances student learning (Garcia-Carrion et al., 2018). There were eight case studies in which student learning outcomes were associated with family skill improvements. It has been found that family education increases students' motivation to learn as their parents attend classes with them (Flecha and Soler, 2014). Family education transforms the learning context at home. Evidence in the reviewed articles shows that school activities involving families increase diversity and provide students with diverse role models (Valls and Kyriakides, 2013; Garcia-Carrion, 2015).

Nineteen peer-reviewed articles demonstrate that PA can reduce absenteeism. By implementing successful practices, schools can strengthen their bonds with their communities, which results in a greater sense of openness on the part of schools toward the local community (Gomez et al., 2014). It has been found that better attendance records are associated with better school attendance (Flecha and Soler, 2014). Therefore, these findings challenge the deficit perspective. Some communities blame insufficient interest in education for absences from school. Considering the reviews, it has been demonstrated that involving parents in the decision-making process results in a more positive relationship between schools and their communities since parents change their perceptions and determine what type of school they want their children to attend (Flecha and Soler, 2014). Implementing effective practices contributes not only to students' individual development but also to their joint development as members of the school community. This reduces absenteeism, which is the primary cause of truancy. Researchers have emphasized the importance of integrating Roma males into schools (Gomez et al., 2014). Consequently, parents do not wish to send their daughters to high school, dispelling another commonly held belief. Therefore, it is evident that SEPs are intricately linked to PE students' success (Wright and Burton, 2008; Beaulac et al., 2011; Connolly et al., 2011; Diez et al., 2011; Karasimopoulou et al., 2012; Noggle et al., 2012; Sifers and Shea, 2013; Standage et al., 2013; Valls and Kyriakides, 2013; Flecha and Soler, 2014; Gomez et al., 2014; Padros, 2014; Aubert, 2015; Garcia-Carrion, 2015; Grace, 2015; Kall et al., 2015; Molina Roldan, 2015; Costigan et al., 2016; Hankonen et al., 2016; Lubans et al., 2016; Smedegaard et al., 2016; Aubert et al., 2017; Bakir et al., 2017; Gül et al., 2017; Ha et al., 2017; Ho et al., 2017; McNamee et al., 2017; Puigdellivol et al., 2017; Capllonch Bujosa et al., 2018; Garcia-Carrion et al., 2018, 2020a,b; Hignett et al., 2018; Valero et al., 2018; Villardon-Gallego et al., 2018; Lopez de Aguileta, 2019; Luna et al., 2019; Slee and Allan, 2019; Villarejo-Carballido et al., 2019; Diez-Palomar et al., 2020; Huescar Hernandez et al., 2020; Khalfaoui et al., 2020; Lopez de Aguileta et al., 2020; Roca et al., 2020; Zubiri-Esnaola et al., 2020). This is because of the promotion of physical literacy, intrinsic motivation, autonomy, and professional development. Overall, these results demonstrate the importance of integrating physical skills into a holistic education program that emphasizes the importance of personal development, well-being, and lifelong participation in PA. PE can result in transformative outcomes for students as educational paradigms evolve and these practices are recognized and harnessed.

## 4. Discussion

PE presents an exciting opportunity for exploration and understanding the intricate relationship between SEPs and student outcomes. Throughout this discussion, we explore the nuances and implications of this relationship, especially for educators, students, and the broader education community. Our analysis shows that effective practices can produce a wide array of effects, and we provide evidence of transformation at different levels. The present research found that participation in successful practices has various effects on students regarding the first goal. Our study had an impact at three levels, not only on the individual, but also on the group and community levels, as shown in Figure 3. In this synthesis, most findings relate to instrumental learning, as most articles focus on student outcomes. Specifically, this refers to improving students' learning abilities regardless of their background. These studies provide evidence of student learning outcomes, from cognitive to purely cognitive, such as test score improvements. In addition, they provide evidence of those that are more effective, such as the development of useful study habits such as perseverance and dedication. Interestingly, this can be attributed to the dialogic learning method (Flecha and Soler, 2014). Collaboration between students and other educational community members is central to dialogic learning. Learning theories emphasize the importance of dialogue in learning. Theoretically, learning and cognitive development are social processes that occur through interaction with others (Vygotsky and Cole, 1978). Through these contacts, new knowledge is created and exchanged (Rogoff, 1990; Wells, 1999). In recent years, numerous studies have suggested that students learn better and more efficiently when dialogue is encouraged between educators and students. Research indicates that initiatives promoting student dialogue enable this type of learning.

SEPs positively influence students' self-esteem and motivation in addition to academic outcomes (Table 1). The current synthesis reports that students who participated in these effective activities reported increased self-esteem, confidence, and future aspirations. It is fascinating to observe that these students come from weaker circumstances. In addition, research has shown that adolescents from low-income families tend to lack self-confidence and have low hopes for the future. However, students can alter their perceptions of themselves when developing positive relationships with peers and other relevant adults (Kim et al., 2014; Khattab, 2015). Therefore, effective strategies were developed in learning communities to assist students who were at risk of not fulfilling their potential. These strategies included improving their self-concept and cultivating better aspirations.

Our current research synthesis revealed that SEPs positively impact relationships among classmates. These impacts include a reduction in conflict, cohesion, and inter-personal relationships. Studies conducted in the literature review indicate that students are more committed to their fellow students' academic success and have a more favorable attitude toward helping one another. In addition, studies have shown that students were less likely to engage in conflict since they were involved in developing their own ground rules for cooperation. This synthesis suggests that students can learn and thrive more easily when these adjustments are made. This is based on the evidence from the studies included in this synthesis. Earlier studies have shown that welcoming and stimulating classroom environments are important for students' academic performance, especially among disadvantaged students (Valero et al., 2018).

It is important to emphasize the importance of peers, relatives, and other members of the community in this synthesis, even though much attention was focused on student results. As a result, the subcategory of family participation and the subcategory of changes in attitude toward school both yielded insight into the outcomes of students. The present synthesis concludes that effective practices and learning communities can help students change their negative attitudes toward school and increase parental involvement. The research included in this study has led to this conclusion. This study found that this change also positively affected students when they observed their parents or other parents participating in school activities, in line with what other researchers have discovered (Clark, 2020). This was because parents volunteered in classrooms, giving students more role models and enhancing their learning experience. This results in students being more motivated to learn and interested in school activities. This study's findings align with research showing the benefits of parental involvement in children's education (Jasis and Ordonez-Jasis, 2012; Jeynes, 2017; Epstein, 2018; Roksa and Kinsley, 2019; Swain and Cara, 2019).

The research analyzed for this synthesis concluded that effective practices and learning communities could reduce absenteeism among pupils. Absenteeism was reduced by implementing successful practices and transforming schools into learning communities. Studies conducted have shown that there is a reciprocal relationship between increasing the participation of the community in schools and increasing the relevance of the school in the community. This reciprocal impact made the students more interested in and committed to school activities. Although few articles have addressed this subject, it is important to note that these studies reflect an era in which absenteeism was very high. In other schools, successful strategies may not be implemented similarly. Several pieces of research examined here demonstrate promising results regarding the effect of effective practices on learners. Despite such restrictions, the authors are aware of them. Comparative studies rely on small, cross-sectional samples, compared to qualitative studies that rely on a single case study. Several problems were also encountered with quasi-experimental studies, such as the necessity of standardizing the initial conditions (Huescar Hernandez et al., 2020), repeating the study in another classroom (Diez-Palomar et al., 2020), and using randomly selected subjects (Valero et al., 2018). SEPs and student outcomes are fundamentally interrelated and transformative. PE that integrates inclusive, technology-driven, and learner-centered strategies not only enhances students' physical skill acquisition but also contributes to their overall development. Aside from laying the foundation for a healthier future, these practices promote life skills such as self-confidence, determination, and an appreciation of PA for a lifetime (Wright and Burton, 2008; Beaulac et al., 2011; Connolly et al., 2011; Diez et al., 2011; Karasimopoulou et al., 2012; Noggle et al., 2012; Sifers and Shea, 2013; Standage et al., 2013; Valls and Kyriakides, 2013; Flecha and Soler, 2014; Gomez et al., 2014; Padros, 2014; Aubert, 2015; Garcia-Carrion, 2015; Grace, 2015; Kall et al., 2015; Molina Roldan, 2015; Costigan et al., 2016; Hankonen et al., 2016; Lubans et al., 2016; Smedegaard et al., 2016; Aubert et al., 2017; Bakir et al., 2017; Gül et al., 2017; Ha et al., 2017; Ho et al., 2017; McNamee et al., 2017; Puigdellivol et al., 2017; Capllonch Bujosa et al., 2018; Garcia-Carrion et al., 2018, 2020a,b; Hignett et al., 2018; Valero et al., 2018; Villardon-Gallego et al., 2018; Lopez de Aguileta, 2019; Luna et al., 2019; Slee and Allan, 2019; Villarejo-Carballido et al., 2019; Diez-Palomar et al., 2020; Huescar Hernandez et al., 2020; Khalfaoui et al., 2020; Lopez de Aguileta et al., 2020; Roca et al., 2020; Zubiri-Esnaola et al., 2020). In light of PE's ongoing evolution, it is crucial to understand and optimize this relationship. Therefore, we are striving to create generations of individuals who are not only physically capable but are also empowered, motivated, and well-rounded.

This comprehensive review identified various knowledge gaps. Additional research will be required to fill in these gaps. First, this research study focused on preschool instruction. Primary school children have more experiences than their kindergarten and preschool counterparts, despite SEPs at all educational levels. Other articles reflect this. Even so, additional research at this academic level would be fascinating to gain further insight into the impact of early success on later life. Second, we observed that some projects received more attention than others, while integrative groups did not receive such attention. Integrative organizations have the most successful practices in the world. Our synthesis focused on the impact on pupils, which could also be one of the reasons why this occurred. This study did not incorporate the findings of other initiatives regarding the impact on the family and community. Despite this, we believe that researchers would benefit from escalating their efforts to investigate other successful practices to better understand their impact. Third, most of the original research for the synthesis was conducted in Spain. Although excellent practice programs and learning communities are widely implemented in other countries, they are underrepresented in peer-reviewed studies. Successful practices and the learning community method are examples of initiatives that schools typically cannot undertake when it comes to conducting research and publishing in academic journals. Conversely, Latin American researchers have limited experience investigating the effects of effective practices and learning communities. Yet, we identified one instance of Spanish researchers and Colombian educators collaborating to implement effective practices and publish their findings in a peer-reviewed journal (Soler et al., 2019). Promoting research in Latin American contexts requires more cooperation of this kind. As for research design, most of the articles examined employed qualitative approaches, although mixed approaches and quantitative approaches were also used to a limited extent (He et al., 2023; Jiang et al., 2023a,b). The extensive use of qualitative methods in articles results from the long tradition of researchers. However, combining quantitative and longitudinal studies would contribute to a deeper and broader understanding of effective practices and learning communities. It is also likely that quasi-experimental studies will expand our understanding of the benefits of effective practices and learning communities on academic and extracurricular performance (Roca et al., 2020).

### 4.1. Limitations

The study has some caveats. First, this literature review focused solely on scholarly articles. Scholarly articles and school administrations (Morla-Folch et al., 2022) support learning communities. These documents provide additional information on effective practices. Second, literature not written in English is not considered. This statistic illustrates academics' growing pressure to communicate their findings in English. Increasing research on adapting effective practices to various contexts is expected to generate insights from highly diverse research teams. This is done without compromising the results quality. Third, our research focused on the role played by effective practices in determining students' final performance. Although effective practices have been extensively studied, this research also focused on these initiatives' impacts on other relevant actors. Volunteers and the entire community were among these actors. Additional literature reviews could explore the impact of productive practices and learning communities outside of the classroom. Fourth, the review sample consisted primarily of students from preschool and primary schools. Although successful techniques have been introduced at other levels of education, they were not included in this review as they were irrelevant to the subject matter.

## 5. Conclusions

This study is relevant to educators, principals, and policymakers in education. Current research summarizes existing data regarding effective student achievement practices. We have found that effective practices enhance learning outcomes for students when accompanied by demanding group activities. Additionally, they ensure that they have the means of discussing and resolving disputes. Emphasizing the importance of all students' perspectives boosts student confidence and drive. These findings are significant for all stakeholders involved in education, as these measures improve school climate, enhance inclusion of all children, and pave the way for dispute resolution. Furthermore, our synthesis demonstrated that successful practices help transform communities, which benefits students. Studies have shown that successful practices contribute to the Sustainable Development Goal of ensuring high-quality education for everyone. Educators at all levels could use this data to inspire positive change in their institutions. The goal is to ensure that all students have access to a high-quality educational environment, regardless of outbreak severity. Literature analysis has demonstrated that promising methods can improve education for low-income students and help social change.

## Data availability statement

The original contributions presented in the study are included in the article/supplementary material, further inquiries can be directed to the corresponding authors.

## Author contributions

JH: Conceptualization, Data curation, Formal analysis, Funding acquisition, Investigation, Methodology, Project administration, Resources, Software, Supervision, Validation, Visualization, Writing—original draft, Writing—review and editing. HY: Data curation, Formal analysis, Methodology, Software, Writing—review and editing. MJ: Data curation, Formal analysis, Methodology, Validation, Writing—review and editing. MB: Project administration, Supervision, Writing—review and editing.
